# Glucagon-Like Peptide-1 Receptor Agonists as Potential Myelination-Inducible and Anti-Demyelinating Remedies

**DOI:** 10.3389/fcell.2022.950623

**Published:** 2022-07-06

**Authors:** Kazunori Sango, Shizuka Takaku, Masami Tsukamoto, Naoko Niimi, Hideji Yako

**Affiliations:** Diabetic Neuropathy Project, Department of Diseases and Infection, Tokyo Metropolitan Institute of Medical Science, Setagaya-ku, Tokyo, Japan

**Keywords:** glucagon-like peptide-1 receptor agonists, neuroprotection, axonal injury, diabetic neuropathy (DN), multiple sclerosis (MS), Schwann cells, olfactory ensheathing cells, oligodendrocytes

## Abstract

Glucagon-like peptide-1 receptor agonists (GLP-1RAs) were developed as insulinotropic and anti-hyperglycemic agents for the treatment of type 2 diabetes, but their neurotrophic and neuroprotective activities have been receiving increasing attention. Myelin plays a key role in the functional maintenance of the central and peripheral nervous systems, and recent *in vivo* and *in vitro* studies have shed light on the beneficial effects of GLP-1RAs on the formation and protection of myelin. In this article, we describe the potential efficacy of GLP-1RAs for the induction of axonal regeneration and remyelination following nerve lesions and the prevention and alleviation of demyelinating disorders, particularly multiple sclerosis.

## 1 Introduction

Glucagon-like peptide-1 (GLP-1) is an incretin hormone secreted from gut enteroendocrine cells in response to food intake, and display insulinotropic actions by stimulating specific G-protein linked GLP-1 receptor (GLP-1R) on the pancreatic β cells. Endogenous GLP-1 is quickly metabolized into the inactive peptide by dipeptidyl peptidase (DPP)-4, whereas GLP-1R agonists (GLP-1RAs) have a substantially longer plasma half-life than GLP-1 because of their resistance to DPP-4. Numerous GLP-1RAs have been developed and utilized in the treatment of type 2 diabetes as injections (liraglutide, exenatide (exendin-4 (Ex-4)), lixisenatide, semaglutide, *etc.*) and oral formulations (semaglutide) (Drucker, 2018). GLP-1Rs are found in not only the pancreas but also the extra-pancreatic tissues, including the central nervous system (CNS) and peripheral nervous system (PNS), and most of the agonists can cross the blood-brain barrier; therefore, neurotrophic and neuroprotective activities of GLP-1RAs have been drawing increasing attention (Harkavyi and Whitton, 2010). Recent studies have suggested the beneficial effects of GLP-1RAs toward neurodegenerative disorders, such as Alzheimer’s disease (AD), Parkinson’s disease (PD), multiple system atrophy, and amyotrophic lateral sclerosis (ALS) (Li et al., 2012; Bassil et al., 2017; Zhang et al., 2021; Holscher, 2022). In particular, liraglutide and Ex-4 significantly mitigated the symptoms and neuropathology of AD and PD in animal models and have provided encouraging evidence in clinical trials for these diseases (Femminera et al., 2019; Esparza-Salazar et al., 2021; Vijiaratnam et al., 2021). The neuroprotective effects of liraglutide and Ex-4 on the PNS have also been documented; these agents facilitated axonal regeneration and functional repair after sciatic nerve injury and ameliorated oxaliplatin-induced and diabetic peripheral neuropathies in rodent models (Himeno et al., 2011; Yamamoto et al., 2013; Fujita et al., 2015; Ma et al., 2018). Recent clinical studies have suggested their efficacy in the treatment of diabetic neuropathy independent of anti-hyperglycemic actions (Wegeberg et al., 2020; Issar et al., 2021). Together, these findings provide further evidence of the direct actions of GLP-1RAs on the CNS and PNS; however, the underlying mechanisms remain largely unclear.

Myelin is the structure that surrounds individual axons and maintains saltatory conduction. In addition, myelin sheaths play a pivotal role in the protection of axons from physical stresses and inflammation. Oligodendrocytes and Schwann cells are myelin-forming cells in the CNS and PNS, respectively. Adequate remyelination of the regenerated axons is a prerequisite for satisfactory functional recovery following peripheral nerve injury (Sango et al., 2017). Previous *in vivo* and *in vitro* studies suggest that Ex-4 accelerates the process of axonal regeneration and remyelination (Yamamoto et al., 2013; Kuyucu et al., 2017; Takaku et al., 2021). In addition, demyelinating disorders (multiple sclerosis (MS), Guillain–Barré syndrome, chronic inflammatory demyelinating polyneuropathy (CIDP), amiodarone-induced neuropathy, *etc.*) are intractable and life-threatening, as well as the neurodegenerative disorders described above. Recent studies have shed light on the therapeutic potential of liraglutide and Ex-4 for demyelinating disorders, mainly MS (DellaValle et al., 2016; Lee et al., 2018; Ammar et al., 2022).

The aim of this review is to characterize GLP-1RAs as favorable factors for myelin formation and maintenance and discuss the possibilities of their repositioning for peripheral nerve injury and demyelinating disorders.

## 2 GLP-1RAs as Favorable Factors for Myelin Formation and Maintenance

### 2.1 ALS Models

There is increasing evidence that impaired GLP-1 signaling is associated with the progression of ALS (Shandilya and Mehan, 2021), and potential efficacy of GLP-1RAs for the disease prevention has been implicated. Li et al. (2012) reported neuroprotective activities of Ex-4 toward SOD1 (G93A) mice, a well characterized animal model of familial ALS; Ex-4 administration via subcutaneous osmotic pump (3.5 pM/kg/min) for 12 weeks attenuated motor neuron death and myelinated nerve fiber loss in the spinal cord of the mutant mice. In contrast, a recent study by (Keerie et al., 2021) showed no significant effects of liraglutide (intraperitoneal injection of 25 or 75 nM/kg/day for 2–6 months) on the disease progression in 2 kinds of ALS model mice, such as SOD1 (G93A) and TDP-43 (Q331K).

### 2.2 Wolfram Syndrome Model

Wolfram syndrome (WS) is an autosomal recessive neurodegenerative disorder characterized by childhood-onset diabetes mellitus and various neurological manifestations, including progressive optic nerve atrophy, sensorineural hearing loss and cognitive impairment (Kabanovski et al., 2022). Seppa et al. (2021) reported that subcutaneous liraglutide injection (0.4 mg/kg/day) on male WS model rats (Wfs1(−/−)) for 3.5 months delayed the progression of optic nerve atrophy and induced remyelination. Because optic nerve fiber degeneration and disruption of myelin sheath integrity appears to be a cause of visual acuity loss in WS, the neuroprotective and myelin restorative activities of liraglutide might be helpful for the maintenance of visual function in WS patients.

### 2.3 Nerve Injury Models

Yamamoto et al. (2013) observed that repeated intraperitoneal injections of Ex-4 (2.5 μg/rat/day) for 14 days was efficacious for the recovery of motor function (sciatic nerve index), electrophysiological data (distal latency), and light and electron microscopic findings (myelinated nerve fiber density and myelin thickness) in rats following sciatic nerve crush injury. Because GLP-1R immunoreactivity at Schwann cells was augmented by Ex-4 (Liu et al., 2011), it seems plausible that Ex-4 promotes axonal regeneration through stimulating Schwann cells mediated by GLP-1R. Kuyucu et al. (2017) examined long-term (12 weeks) effects of subcutaneous Ex-4 injections (10 μg/rat/day) on rats following sciatic nerve transection. Ex-4 administration improved muscle strength of hindlimbs, electrophysiological data (latency and amplitude), and nerve fiber density; however, its effects on myelinated nerve fiber density and myelin thickness were not documented. These findings imply promising effects of Ex-4 and other GLP-1RAs on remyelination of the regenerated axons following nerve injury, although the precise action mechanisms remain to be determined.

### 2.4 Diabetic Neuropathy Models

There is enough evidence that the main pathology of diabetic neuropathy is axonal degeneration, which precedes demyelination observed in patients with the disease at advanced stages (Niimi et al., 2021). However, Schwann cell apoptosis and de-differentiation under diabetic conditions might contribute to myelin thinning and derangement (Hao et al., 2015; Naruse, 2019). Several studies have been devoted to the ameliorating effects of GLP-1RAs on reduced myelinated nerve fibers and/or Schwann cell abnormalities in streptozotocin (STZ)-induced diabetic rats. Intraperitoneal injections of Ex-4 (1 nmol/kg/day) for 24 weeks restored myelinated fiber size and prevented Schwann cell apoptosis in STZ-diabetic rats (Liu et al., 2011). Similarly, intraperitoneal injections of liraglutide (200 μg/kg/day) for 8 weeks improved the delayed motor and sensory nerve conduction velocities and reduced myelinated nerve fiber density in STZ-diabetic rats (Ma et al., 2018). Furthermore, treatment of STZ-diabetic rats with a new synthetic arginine-rich Ex-4 (Peptide D, 0.1–10 μg/kg/day) for 80 days ameliorated neuropathic pain and reduced myelinated nerve fiber diameters and myelin basic protein (MBP) expression in sciatic nerves (Shekunova et al., 2020). These findings suggest the favorable effects of GLP-1RAs on the maintenance of myelin structure and function in the diabetic neuropathy model. Because neither Ex-4 nor liraglutide normalized the blood glucose levels of diabetic rats, these agents are likely to exert myelin-protective activities through direct actions on neurons and Schwann cells rather than anti-hyperglycemic actions.

### 2.5 Neuron–Schwann Cell Co-Culture Models

**FIGURE 1:**
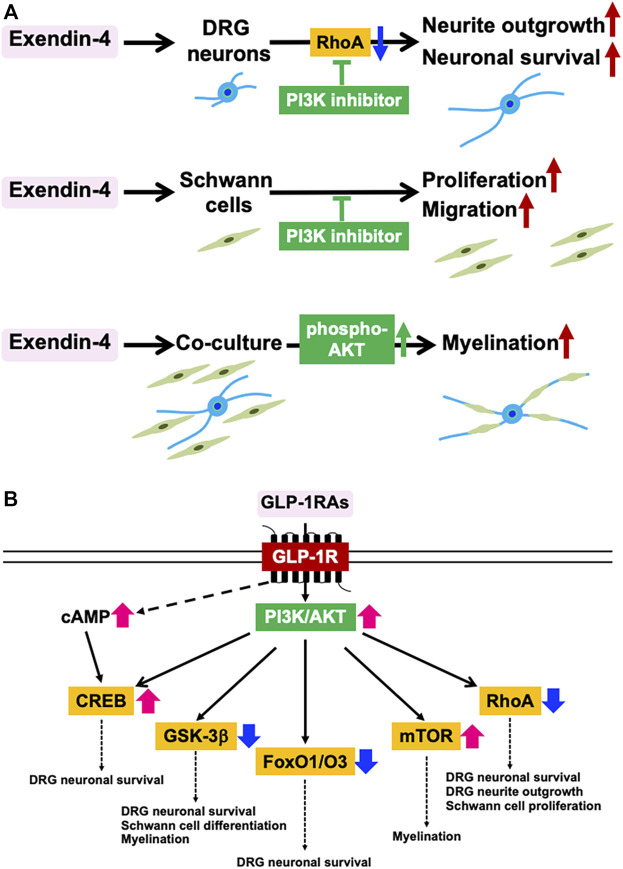
Myelination-inducible activities of GLP-1RAs mediated by PI3K/AKT signaling pathway. **(A)** Involvement of PI3K/AKT signaling pathway in the favorable effects of Ex-4 on DRG neurons, IFRS1 Schwann cells, and their co-culture system in the authors’ studies (Tsukamoto et al., Histochem. Cell Biol. 2015; Takaku et al., Int. J. Mol. Sci. 2021). **(B)** Downstream molecules of GLP-1R-PI3K/AKT pathway and their potential roles in myelination. CREB; cAMP response element-binding protein, FoxO1/O3; Forkhead box O1/O3, GSK-3β; glycogen synthase 3β, mTOR; mammalian target of rapamycin (Modified from Athauda & Foltynie, Drug Discov. Today 2016).

Co-culture systems of neurons and Schwann cells have enabled us to generate myelin structures nearly equivalent to those in living bodies, thereby being recognized as useful tools to investigate neuron-Schwann cell interactions (Ogata et al., 2004). Immortalized adult Fischer rat Schwann cells 1 (IFRS1) established in our laboratory display distinct Schwann cell phenotypes, including fundamental ability to myelinate neurites in co-culture with adult rat dorsal root ganglion (DRG) neurons (Sango et al., 2011), nerve growth factor-primed PC12 cells (Sango et al., 2012), and NSC-34 motor neuron-like cells (Takaku et al., 2018). As compared with the previous co-culture models using embryonic and/or neonatal animals, our DRG neuron-IFRS1 co-culture system has the following advantages: 1) Both neurons and Schwann cells are derived from adult rats and retain the biological properties of the mature peripheral nervous system. 2) Immortalized Schwann cells can be stably and effectively utilized in co-culture. In our recent study, Ex-4 applied to culture medium (100 nM) accelerated myelination process in the co-culture system with activation of serine/threonine-specific protein kinase AKT (Takaku et al., 2021). Ex-4 enhanced the movement of IFRS1 cells toward the neurites and upregulated the protein expression of peripheral myelin protein 22 (PMP22) and myelin protein zero (MPZ). The existence of GLP-1R in both DRG neurons and IFRS1 Schwann cells was confirmed by knock-out validated anti-GLP-1R antibody (Takaku et al., 2021), and Ex-4 promoted neurite outgrowth of DRG neurons (Himeno et al., 2011; Tsukamoto et al., 2015) and survival/proliferation and migration of Schwann cells (Pan et al., 2020; Takaku et al., 2021). These findings suggest that Ex-4 stimulates GLP-1R in both DRG neurons and Schwann cells to promote myelination. Establishment of GLP-1R-deleted IFRS1 Schwann cells (Takaku et al., in preparation) will further confirm this hypothesis. Because Ex-4 induced AKT phosphorylation in the co-culture and its beneficial effects on DRG neurons and IFRS1 cells were attenuated by phosphatidyl inositol-3′-phosphate-kinase (PI3K) inhibitor LY294002, the myelination-inducible activities of Ex-4 may be attributable to the activation of PI3K/AKT signaling pathway in both cells (Figure 1A). Although the stimulation of GLP-1R has been shown to activate PI3K/AKT pathway in the PNS (Tsuboi et al., 2016; O’Brien et al., 2019) and this pathway appears to be crucial for initiating myelination (Ogata et al., 2004), which downstream molecules and pathways are more involved in the Ex-4-induced myelination remain to be proved. The neuroprotective activities of GLP-1RAs against PD can be, at least partially, mediated by PI3K/AKT pathway that modulates several downstream molecules, such as cAMP response element-binding protein (CREB), glycogen synthase 3β (GSK-3β), Forkhead box O1/O3 (FoxO1/O3), and mammalian target of rapamycin (mTOR) (Athauda and Foltynie, 2016) (Figure 1B). Among these molecules, activation of CREB and inhibition of GSK-3β and FoxO1/O3 are likely to participate in the neuroprotective activities of insulin, IGF-1, and GLP-1 toward DRG neurons (Leinninger et al., 2004; Tsuboi et al., 2016). GSK-3β inhibition is also involved in Schwann cell differentiation and myelination (Ogata et al., 2004). By using insulin receptor-deleted Schwann cells, Hackett et al. (2020) indicated a pivotal role of PI3K/AKT/mTOR signaling in myelination. Besides these molecules, RhoA inhibition through PI3K/AKT pathway was shown to stimulate survival and neurite outgrowth of DRG neurons (Tsukamoto et al., 2015) and proliferation of Schwann cells (Tan et al., 2018).

Although there have been no reports regarding the efficacy of GLP-1RA for oligodendrocyte function *in vitro*, growing evidence with Schwann cells may be applicable to CNS myelination in the future.

### 2.6 Culture of Olfactory Ensheathing Cells (OECs)

OECs are the glial cells of the primary olfactory system, and its transplantation is a promising strategy for functional repair following spinal cord and peripheral nerve injuries (Chou et al., 2014). Tseng et al. (2021) observed stimulatory effects of liraglutide (100 nM) on the migration of primary cultured and lined OECs with activation of the extracellular signal-regulated kinases (ERK) pathway and modulation of extracellular matrix proteins (upregulation of laminin-1 and downregulation of type IV collagen). These findings imply the potential efficacy of liraglutide and other GLP-1RAs for improving OECs transplantation outcomes, including remyelination.

## 3 Potential Efficacy of GLP-1RAs Toward Demyelinating Disorders

### 3.1 Multiple Sclerosis (MS)

There are more than 2 million patients with MS worldwide. Like AD and PD, MS can be categorized into neurodegenerative disorders in the CNS; however, it has distinct features of progressive immune-mediated neuro-inflammatory evens resulting in multiple demyelinating lesions (Yamasaki and Kira 2019). Although tremendous efforts have been made on the achievement of myelin repair and neuroprotection in MS, its complex pathogenesis obstacles satisfactory outcomes for the patients with progressive phase (Sandi et al., 2022).

It is recognized that immune cells play a major pathological role in MS; they disrupt myelinated axons and evoke demyelination and oligodendrocyte cell death. To better understand the pathology of demyelination in MS, several animal models have been established; in particular, experimental autoimmune encephalomyelitis (EAE), and cuprizone-induced demyelination models are widely used (Bando, 2019). The potential efficacy of GLP-1RAs toward MS has been documented in recent studies with EAE models, but their activities are mostly restricted to the modulation of immune reactions. For instance, intraperitoneal Ex-4 injection (5 μg/kg/day) for 13 days to EAE mice reduced the clinical symptoms, histopathological sequelae (demyelination, astrogliosis, and microglial activation), and mRNA expression of proinflammatory cytokines (IL-1β, IL-6, IL-17, and TNF-α) (Lee et al., 2018). Likewise, subcutaneous dulaglutide injection (180 μg/kg/twice per week) to EAE mice attenuated the clinical manifestations and histopathological outcomes (lymphocyte infiltration, vacuolar degeneration, and neuronal demyelination), as well as decreased incidences of encephalitogenic Th1/Th17 cells and Th1 granulocyte-macrophage-colony-stimulating factor expression in the CNS (Chiou et al., 2019). Therapeutic potential of liraglutide and a novel GLP-1RA, NLY_01_, in EAE mice has been documented (Gharagozloo et al., 2021; Song et al., 2022), although their action mechanisms seem to be like those of Ex-4 and dulaglutide described above. It is of interest to note that subcutaneous liraglutide injection (200 μg/kg/twice-daily) to EAE rats for 2 weeks reduced the clinical debut and severity and increased the mitochondrial manganese superoxide dismutase (MnSOD) in the brain (DellaValle et al., 2016). These findings imply the capacity of GLP-1RAs against oxidative stress. In a recent study (Ammar et al., 2022), intraperitoneal injection of liraglutide (25 nmol/kg/day) to cuprizone-induced MS model mice for 4 weeks improved the behavioral profiles and remyelination process through stimulating oligodendrocyte progenitor cell differentiation. To our knowledge, this is the first study to introduce GLP-1RAs as oligodendrocyte-protective molecules. Because the expression of GLP-1R in myelin-forming oligodendrocytes has been implicated (Smith et al., 2022) and the stimulatory effects of GLP-1RAs on Schwann cells and OECs have been delineated (Pan et al., 2020; Takaku et al., 2021; Tseng et al., 2021), it seems plausible that the agents can directly act on oligodendrocytes and their progenitor cells to promote remyelination under MS conditions.

### 3.2 Other Demyelinating Disorders

As far as we searched, no studies have ever tried to investigate the potential efficacy of GLP-1RAs for demyelinating disorders other than MS (Guillain–Barré syndrome, CIDP, combined central, and peripheral demyelination, *etc.*). However, accumulating evidence regarding their utility toward MS might be diverted to those diseases. The dual effects of GLP-1RAs, such as modulatory actions to immune cells (lymphocytes, microglia, astrocytes, *etc*) and trophic and protective activities to oligodendrocytes and Schwann cells, would be variable for the strategies of myelin protection and repair in the CNS and PNS. Immune checkpoint inhibitors (ICIs), a novel class of antineoplastic remedies, have shown clinical efficacy toward a variety of intractable tumors. However, ICIs can evoke demyelinating disorders in the CNS (Oliveira et al., 2020) and PNS (Okada et al., 2021) as neurological immune-related adverse events (nirAE). Although demyelination induced by ICIs should be promptly diagnosed and treated according to the guideline for each specific disease (e.g., MS), prescription of GLP-1RAs might be efficacious for the nirAE as a concomitant therapy with immunosuppressors (corticosteroids, intravenous immune globulin, plasmapheresis, *etc*) (Rajendram et al., 2021).

## 4 Conclusion

We briefly summarized the recent progress regarding the beneficial effects GLP-1RAs on myelin formation and maintenance and their potential efficacy toward MS and other demyelinating disorders. The underlying action mechanisms remain largely obscure, and no clinical trials of GLP-1RAs toward axonal injury or MS have been conducted; however, the broad distribution of GLP-1R in the nervous tissue and diverse biological activities of GLP-1RAs (Drucker, 2018; Holscher, 2022) would enable their repositioning for those disorders. Considering the utility and safety of GLP-1RAs as existing anti-diabetic remedies (Gilbert et al., 2020) and the advancement of clinical trials of Ex-4 toward PD (Vijiaratnam et al., 2021), it does not seem exaggerated to describe them as promising agents for myelin regeneration and repair.
